# The use of infrared thermography to detect the stages of estrus cycle and ovulation time in anatolian shepherd dogs

**DOI:** 10.1186/s40781-017-0146-4

**Published:** 2017-10-09

**Authors:** Kemal Tuna Olğaç, Ergun Akçay, Beste Çil, Burak Mehmet Uçar, Ali Daşkın

**Affiliations:** 1 Department of Reproduction and Artificial Insemination, Faculty of Veterinary Medicine, Ankara University, 06110 Dışkapı/Ankara, Turkey; 2 Diyarbakır Hippodrome, Race Horse Hospital, Economic Establishment of Horse Breeding and Racig of Turkey Jockey Club Association, 21750 Çınar/Diyarbakır, Turkey

**Keywords:** Bitch, Estrus, Estrus detection, Ovulation, Thermography

## Abstract

**Background:**

The aim of the study is to evaluate the effectiveness of thermographic monitoring, using the temperature changes of perianal and perivulvar areas for the determination of estrus in Anatolian Shepherd bitches. Fifteen bitches were used in the study. Blood and vaginal smear samples were collected and thermographic monitoring of perianal and perivulvar areas were carried out starting from proestrus to early diestrus. Also, external signs of estrus were investigated. Smear samples were evaluated by light microscopy after Diff-Quik staining method and superficial and keratinized superficial cells were determined as percentage (S + KS%). Progesterone and luteinizing hormone measurements were done by radioimmunoassay. The difference in temperature between perianal and perivulvar areas was evaluated through thermographic images by FLIR ResearchIR Software.

**Results:**

According to the results obtained from the study, differences between progesterone and S + KS% were statistically significant (*P* < 0,05). Although temperature showed increase and decrease with progesterone and S + KS%, the differences were not important statistically (*P* > 0,05). Serum luteinizing hormone levels did not sign any difference (*P* > 0,05).

**Conclusions:**

As a result, thermographic monitoring alone is not enough for estrus detection in Anatolian Shepherd bitches. However, it can be used to assist the actual estrus detection technique in terms of providing some foreknowledge by evaluating the differences in temperature.

## Background

Ultrasound, endoscopy, vaginal citology, and blood hormone analysis are used effectively to detect the estrus and ovulation time in bitches. These methods have been optimized thanks to numerous studies and they can reveal successful results. Although true results are obtained, these methods are invasive and expensive. Since the procedure is long, bitches have to be stable for a long period. This kind of problems revaled the necessity for new, fast, easy and non-invasive methods for estrus detection.

In bitches, the most accurate results for defining the stages of the estrus cycle and optimal insemination time could be achieved by vaginal cytology and measuring blood progesterone (P4) concentration. Vaginal epithelium is composed of stratified squamous epithelium and these cells undergo metamorphosis with the effect of gonadal hormones in bitches. These differences provide advantages to define cycle stages and ovulation time according to cell characterization and visibility of cell types that appear on smear [1, 2]. Differentiation of vaginal epithelium shows parallelism with the increase and decrease in the concentration of reproductive hormones. Estrogen is one of the important hormones of the cycle. Although concentration of estrogen in blood does not inform us about the exact time of ovulation, its effects on reproductive cells and tissues reveal important signs for the stages of estrus. Estrogen affects vaginal cells and cause them to proliferate and grow (basal cells transform into superficial cells), they lose their vital feature and are keratinized. Also, the vaginal epithelium becomes thicker and gyroser. Once estrogen reaches the peak at the last one-third of proestrus, it begins to decrease. Progesterone begins to increase at the same time with the estrogen peak and the rest of the cyclic events develop under the control of progesterone. Therefore, progesterone is the most important sign for detecting the estrus and ovulation time. In this period, vaginal cytology is characterized with superficial and keratinized superficial cells which are dominant in the chart [1, 3, 4]. Daily blood sampling and vaginal cytology analysis are obligatory for bitches because of daily, even hourly, variations in hormones and the stages of cells [5].

The success of monitoring techniques, such as endoscopy and ultrasonography (USG), for detecting the estrus and ovulation time has been tested in various studies [6, 7]. Variations in the color of vaginal mucus and fold widths of vagina have been monitored with vaginal endoscopy and exact results have been obtained [6]. Determination of cyclic periods and follicular and luteal structures transabdominally by B-mode and doppler USG monitoring, and differences in ovarian blood flow relating with cyclic periods are effective in detecting the estrus and ovulation time [7, 8]. Although these methods are non-invasive, monitoring can be difficult because animals have to be restrained for a long period during the examination.

Thermography is a current diagnostic innovation, for human and animal health, that can detect temperature as infrared radiation spreads from the body surface. Abnormal termal images that can be monitored from skin point to superficial inflamations or alteration in blood flow [9]. It has been used for diagnosing some diseases that cause an increase in superficial temperature, such as laminitis and mastitis [10–12]. Furthermore, it has been found out that there is a relation between scrotal skin temperature and testicular function [13–15]. In recent years, thermographic monitoring has become a useful technique and provided valuable results in animal reproduction. It has been used for detecting estrus and estrus stages in cows, sheep, goats and pigs [16–19]. According to results of these studies [16–19], the difference in temperature between the body and the vulvar surface has begun to increase at proestrus and estrus stages. At the onset of estrus, this difference is at the highest level and then it decreases till the ovulation time.

Although this technique has been studied in various animal species, it has not been tried in dogs yet. The aim of this study is to compare the current estrus detection techniques with infrared thermography. This study has been designed to evaluate the success of infrared thermography in estrus detection and to collect information to create a protocol by comparing temperatures of perivulvar and perianal regions in Anatolian Shepherd bitches through infrared thermography. Furthermore, thermographic monitoring, which is used alone or with other current estrus detection applications, will provide benefits for science and clinical reproduction.

## Methods

### Animal material

Bitches were examined and the healthy ones in terms of general and reproductive health were included into the study program. Fifteen Anatolian Shepherd bitches that were 2–5 years of age were used in the study.

### Study methods

Bitches were involved in the study when proestrus bleeding was first detected. The first day was called Day0 and vaginal smears and blood samples were collected once in 2 days until Day20. Perianal and perivulvar areas were monitored thermographically. Also, the bitches were observed for external signs of estrus such as vaginal bleeding, tail lifting, vulvar edema and vulvar hiperemia. External signs were evaluated daily to regulate the study program.

### Vaginal smear

The dogs were fixed in standing position to collect vaginal smear samples. Epithelial cells were obtained from the anterior side of the vagina wall using swabs humidified with serum physiological. After the samples were transferred to a clean slide by rolling the swab on the long side of the slide, they were dried for 5–10 s in the air and stained with the Diff-Quick by soaking in A solution for 10 s, B solution for 6 s and C solution for 6 s respectively. The stained preparate was washed with distilled water and dried in the air. Later, all smear samples were evaluated under the light microscopy. The percentage of basal-parabasal (B), intermediary (I), superficial (S) and keratinized superficial (KS) cells was determined by counting 200 cells for every slide.

### Hormone analysis

Blood samples collected from the animals were centrifuged as 3000 rpm for 10 min for the purpose of separating blood serum. Levels of luteinizing hormone (LH) and progesterone (P4) were determined from the serum. Hormone levels were measured by radioimmunoassay technique in Sekans Hayvan Sağlığı Laboratuvar Danışmanlık Hizmetleri SAN. LTD. ŞTİ (Ankara, Turkey).

### Termography

The dogs were fixed in standing position for thermographic monitoring. Images were taken 1–1,5 m away from the back side of the bitch. Also, images were taken in a way that they included perianal and perivulvar areas by FLIR e60 Infrared Camera. Images were analyzed with FLIR ResearchIR Software (V 4.20.2.74) and average differences in temperature between perianal and perivulvar areas (Δ°C) were determined on the same day for each dog.

### Statistical analysis

Before the materiality test, all variants have been evaluated with the Shapiro-Wilk Test in terms of parametric test hypothesis and the Levene Test in terms of variance homogeneity. Differences with the control groups have been evaluated with the Kruskal Test because of the data that could not provide normal distribution and the hypothesis of variance homogeneity. Minimum 5% tolerance has been accepted for all statistical analyses. SPSS 14.01 software programme has been used.

## Results

**Fig. 1:**
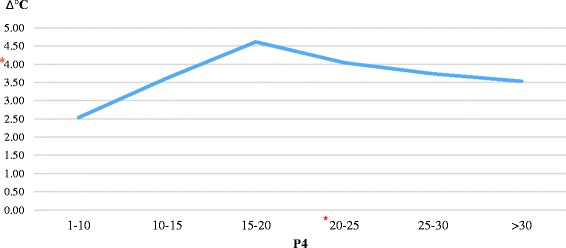
Δ°C quantities depend on P4 amount. *: Indicates the P4 and Δ°C values which ovulations take place

**Fig. 2:**
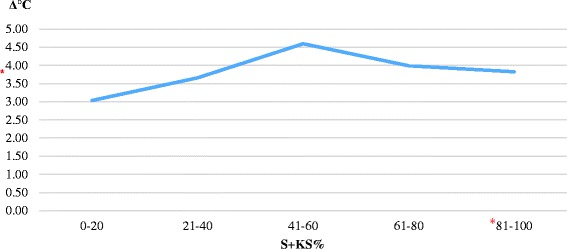
Δ°C quantities depend on S + KS% amount. *: Indicates the S + KS% and Δ°C values which ovulations take place

The difference in the percentage of superficial (S) and keratinized superficial (KS) cells (S + KS%) with serum progesterone (P4) level is statistically significant (*p* < 0,05). Similarly, KS% rates also showed a significant difference with the serum progesterone (P4) level. When the serum P4 level was 20–25 ng/ml, KS% and S + KS% rates were observed in peak values (42,86% ± 6,95; 78,57% ± 5,04) (*p* < 0,05). When KS% and S + KS% rates continued to decrease, the serum P4 level continued to increase. The average P4 amount is 23,98 ± 1,76 ng/ml, when S + KS% was found at the top level (81–100%) (*p* < 0,05). Although the difference in average temperature of perianal and perivulvar areas (Δ°C) showed an increase and decrease as related to P4 and S + KS%, this difference was not statistically significant (*p* > 0,05). The serum LH level was 0,54 ± 0,03 mIU/ml throughout the study (*p* > 0,05). Results were shown in Tables 1 and 2 and variations of Δ°C that depend on estrus stages were shown in Figs. 1 and 2.

**Table 1:** KS%, S + KS%, Δ°C and serum LH quantities depend on P4 amount (blood flow) (X±SE)

P4 (ng/ml)	KS (%)	S + KS (%)	Δ (°C)	LH (mIU/ml)	Ovulations
1–10	10 ± 1,97^c^	29,5 ± 6,47^c^	2,54 ± 0,37	0,55 ± 0,01	-
10–15	17 ± 5,36^bc^	44,67 ± 7,61^bc^	3,62 ± 0,51	0,55 ± 0,01	-
15–20	35,33 ± 7,68^ab^	67,67 ± 7,02^a^	4,61 ± 0,59	0,54 ± 0,01	-
20–25	42,86 ± 6,95^a^	78,57 ± 5,04^a^	4,04 ± 0,64	0,54 ± 0,01	+
25–30	37,78 ± 9,40^ab^	73,89 ± 10,92^a^	3,74 ± 0,68	0,54 ± 0,01	-
>30	35,71 ± 11,10^ab^	57,86 ± 13,97^ab^	3,53 ± 0,58	0,54 ± 0,01	-

^a, b, c^: Averages in groups in the same row with different superscripts are statistically important (*p* < 0,05)

**Table 2:** P4, KS%, Δ°C and serum LH quantities depend on S + KS% in vaginal smear (X±SE)

S + KS (%)	P4 (ng/ml)	KS (%)	Δ (°C)	LH (mIU/ml)	Ovulations
0–20	14,90 ± 2,67^b^	7,14 ± 1,62^b^	3,04 ± 0,47	0,55 ± 0,01	-
21–40	16,09 ± 3,43^b^	12,27 ± 2,01^b^	3,65 ± 0,51	0,55 ± 0,01	-
41–60	17,76 ± 1,87^ab^	23,33 ± 4,97^ab^	4,60 ± 0,82	0,53 ± 0,01	-
61–80	19,81 ± 2,05^ab^	23,46 ± 7,55^ab^	3,99 ± 0,72	0,54 ± 0,01	-
81–100	23,98 ± 1,76^a^	59,57 ± 6,23^a^	3,82 ± 0,39	0,54 ± 0,01	+

^a, b^: Averages in groups in the same row with different superscripts are statistically important (*p* < 0,05)

## Discussion

This study has been designed to evaluate the success of the use of infrared thermography in estrus detection and to collect information to create a protocol by comparing temperatures of perivulvar and perianal regions in Anatolian Shepherd bitches with infrared thermography. Thermography is a current diagnostic innovation, for human and animal health, that can detect temperature as infrared radiation spreads from the body surface. This is an original idea for dog species and Anatolian Shepherd dogs due to the use of thermography in estrus and ovulation time detection through the changes in temperature in perivulvar region. Furthermore, thermographic monitoring, used alone or with other current estrus detection applications, will provide some benefits as an easy technique for science and clinical reproduction.

Nowadays, vaginal smear analysis is the easiest technique that is used for the detection of estrus and prediction of insemination time in bitches. Although vaginal smear analysis is easy, it could provide only basic information about the stages of estrus. This technique needs to be supported with the serum P4 level, because the aspects of vaginal smear are similar between the transition from proestrus to estrus or estrus to diestrus. Therefore, these techniques should be used together. The amount of estrogen in blood increases with proestrus. Once it reaches the peak level in the last 3 days of proestrus, it begins to decrease. LH reaches the peak level in 24–48 h after the estrogen peak. Therefore, LH surge indicates that ovulation will begin in 24–48 h. The percentage of superficial and keratinized superficial cells is 60–80% in vaginal smear. Progesterone, which is below 1 ng/ml until late proestrus, increases with the estrogen peak. During the LH surge, it increases to 2–2,5 ng/ml, and on the day when the ovulation begins progesterone reaches 4 ng/ml. Ovulation continues until the progesterone level exceeds 10 ng/ml [1, 3, 4]. In this stage, the percentage of superficial and keratinized superficial cells is 80–100 in vaginal smear. When the level of progesterone continues to increase at the beginning of diestrus, the percentage of cells decreases [2].

In this study, P4 levels obtained during the estrus cycle did not show consistency with the literature, but it can be seen that P4 courses showed parallelism with the physiology. According to these results, the average P4 level was measured as 23,98 ± 1,76 (*p* < 0,05) when the rate of superficial and keratinized superficial cells was maximum (81–100%) in bitches. Although the serum P4 level was higher in estrus than mentioned in the literature, its course throughout the estrus is acceptable physiologically. It can be seen in Table 2 that the P4 level has a correlation with S + KS% variance. Maximum S + KS% rate average (78,57% ± 5,04), was obtained when the P4 level was 20–25 ng/ml (Table 1). Table 1 shows that S + KS% increases with the P4 level first and after it reaches its surge level (78,57% ± 5,04), it begins to decrease (*p* < 0,05). Although obtained results do not match with the literature in terms of P4 level, the estrus process supports the reproductive physiology in bitches. It is difficult to say the exact time of ovulation via progesterone and LH values. Despite, when cross-examination is made between progesterone and the cell percentages (Tables 1 and 2), it can be seen that the ovulations take place when progesterone is between 20 and 25 ng/ml, because of the highest S + KS% values. Smilarly when maximum S + KS% level is observed, progesterone level is almost 24 ng/ml.

The LH level was around 0,54 ± 0,03 mIU/ml throughout the study. In bitches, LH shows a surge only once in a short period. This rapid increase and decrease of LH takes 24–40 h [20]. The LH peak could not be detected because blood samples were taken once in every 48 h. Furthermore, due to the fact that the other hormone tests were also resulted with surprising data, it can be thought that Anatolian Shepherd bitches may have different LH releasing traits.

In recent years, thermographic monitoring became a useful technique and provided valuable results in animal reproduction. It was used to detect estrus and cycle stages in cows, sheep, goats and pigs [16–19]. According to these studies, the temperature in the vulvar area decreases during the proestrus period. Due to the estrogenic effect, blood flow in genital organs accelerates and edema occurs in vulva throughout the proestrus and at the onset of the estrus; thus, vulvar temperature decreases. Then it begins to increase again in the estrus period and ovulation time. Reducing estrogenic effect in ovulation time causes lower vulvar edema and vulvar temperature increases again [16, 18].

Although the difference between perianal and perivulvar temperatures (Δ°C) were not statistically significant, it was similar with the results of other studies. Δ°C is 2,5–3,5 °C at the onset and in the middle of proestrus; then it reaches 4,6 °C at the end of proestrus and at the onset of estrus. Δ°C decreases to 4 °C in the estrus period, when it can be called as ovulation time according to the percentage of superficial and keratinized superficial cells. After this highest percentage of cells, P4 continued to increase and S + KS% and Δ°C decreased correspondingly. This study was carried out in three different seasons (February–July) and thermographic monitoring was done out of doors. Various environmental situations, such as the different regions and climatic conditions of Turkey may have caused that thermographic findings of the study are not statistically significant, although the results for Anatolian Shepherd bitches are similar with other results.

## Conclusions

In conclusion, thermographic monitoring alone is not enough for detecting the stages of estrus and predicting insemination time in Anatolian Shepherd bitches. However, it could be used together with other current techniques. For example, current techniques such as vaginal smear analysis or P4 measurement can be started when Δ°C is 4,6 °C. The situation when Δ°C is 4,0 °C requires more attention because it is possible that the bitch is at the proestrus or insemination time. Thus, Δ°C needs to be followed in terms of increase or decrease and the estrus detection process should be managed according to these variations. The results of this study have been evaluated only for Anatolian Shepherd bitches. It is essential that these results should be tested on more animals to reach exact data and to facilitate the estrus cycle. In addition, the use of infrared thermography can not only provide general information for dog breeds but also decrease the cost and time spent for detecting estrus on other dog breeds. Futhermore, the reliability and accuracy of thermographic monitoring technique can improve with more studies in an isolated environment from various external conditions and also subsequent studies should be in the same conditions.

## Abbreviations

B: Basal-parabasal cell
I: Intermediary cell
KS: Keratinized superficial cell
LH: Luteinizing hormone
P4: Progeterone
S: Superficial cell
USG: Ultrasonography
Δ°C: Differences in temperature between perianal and perivulvar areas
